# Supplementary material for: Allantoin ameliorates chemically-induced pancreatic β-cell damage through activation of the imidazoline I3 receptors
Source: PeerJ. 2015 Aug 6;3:e1105. doi: 10.7717/peerj.1105 (PMC4540048; doi:10.7717/peerj.1105)

## Slide 1
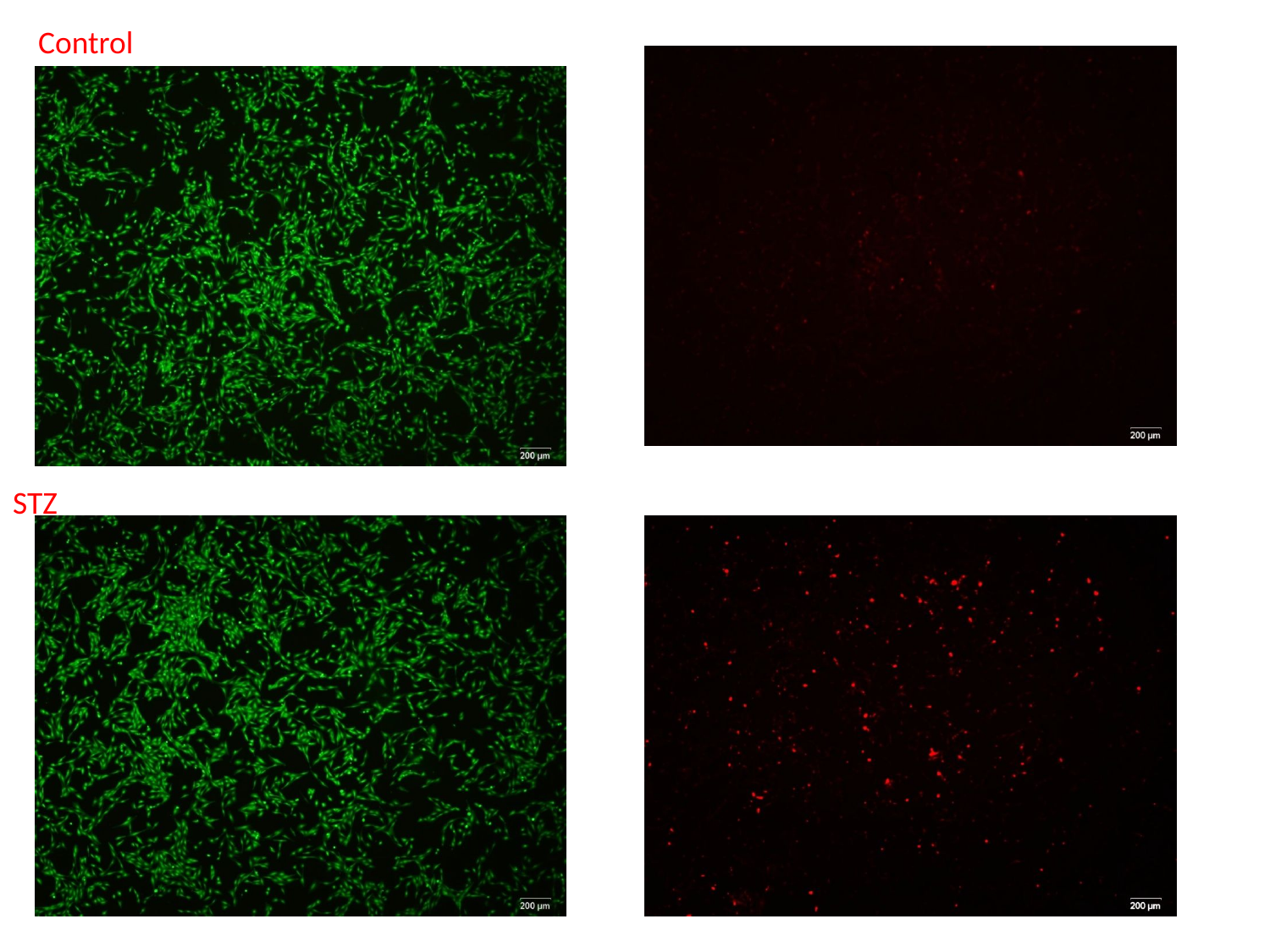

Control
STZ

## Slide 2
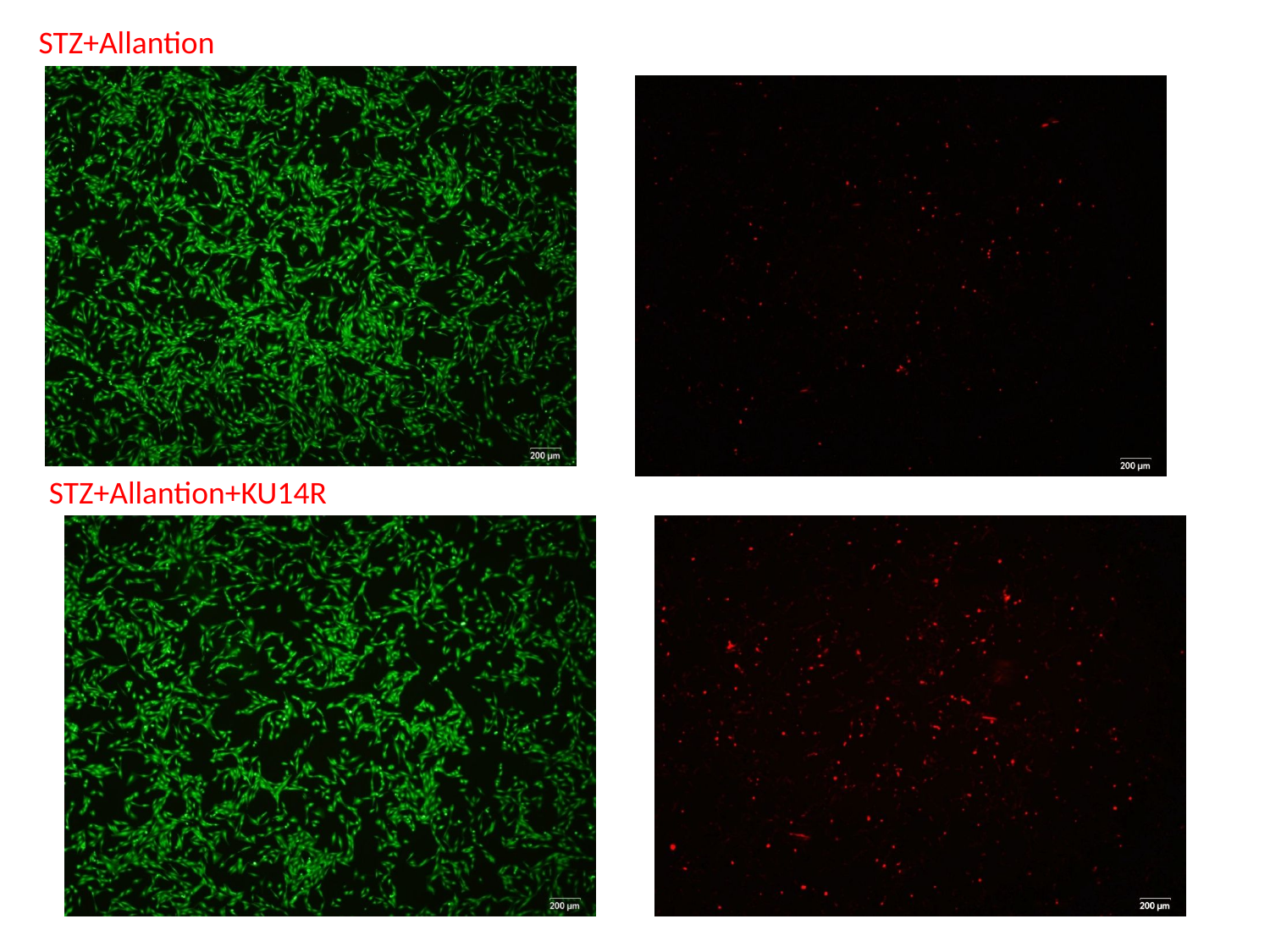

STZ+Allantion
STZ+Allantion+KU14R

## Slide 3
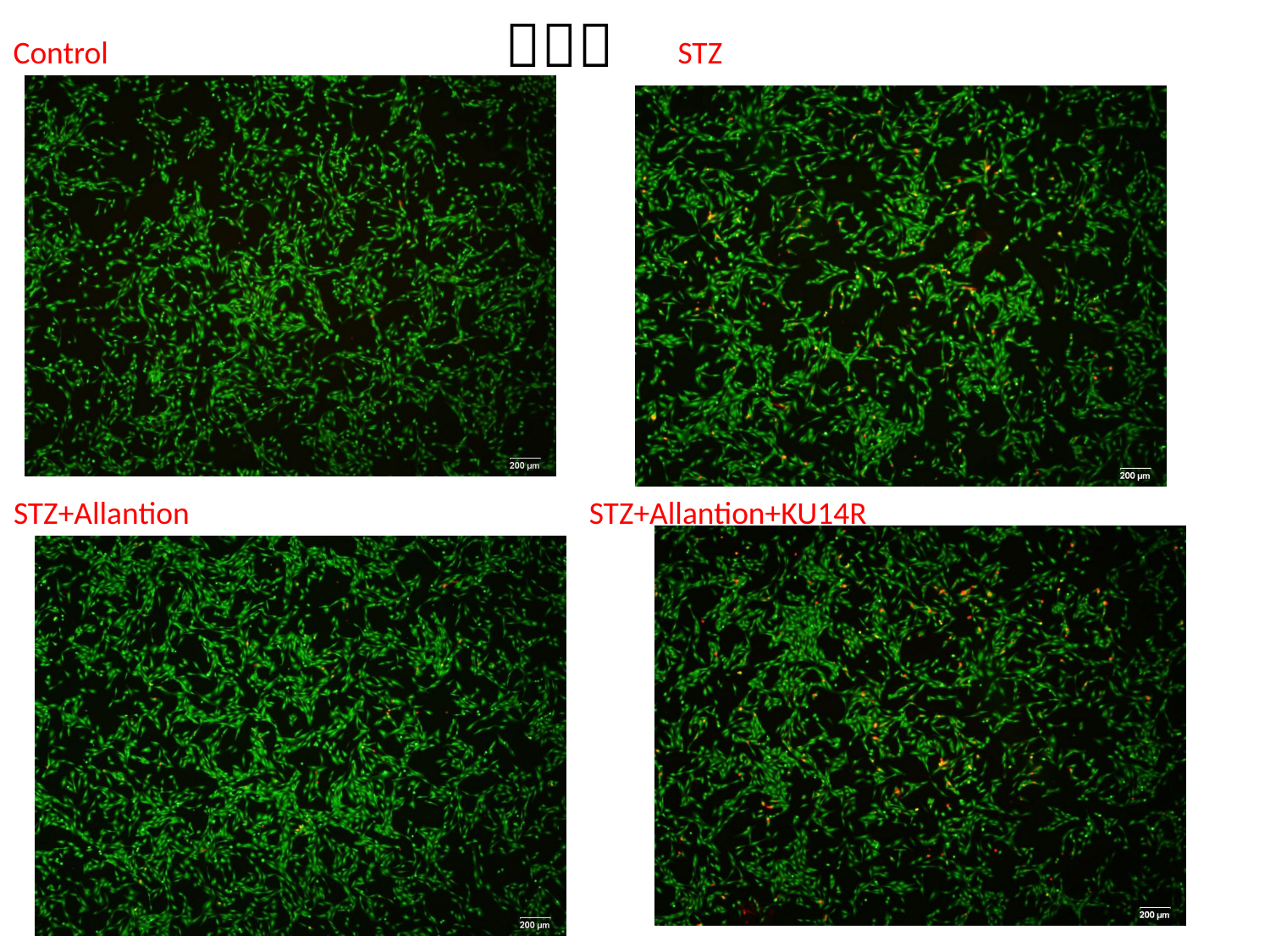

# 合併後
Control
STZ
STZ+Allantion
STZ+Allantion+KU14R

## Slide 4
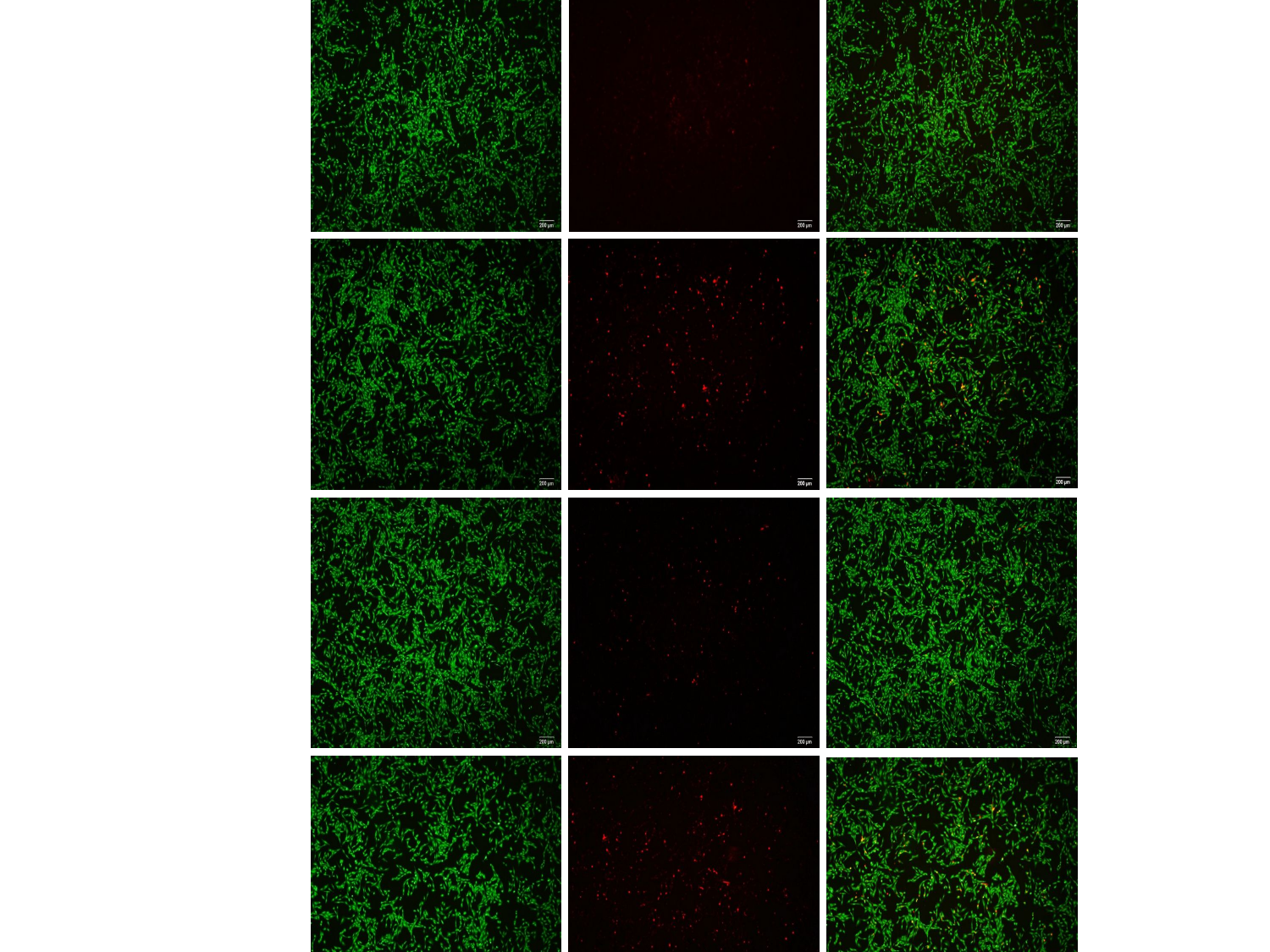

## Slide 5
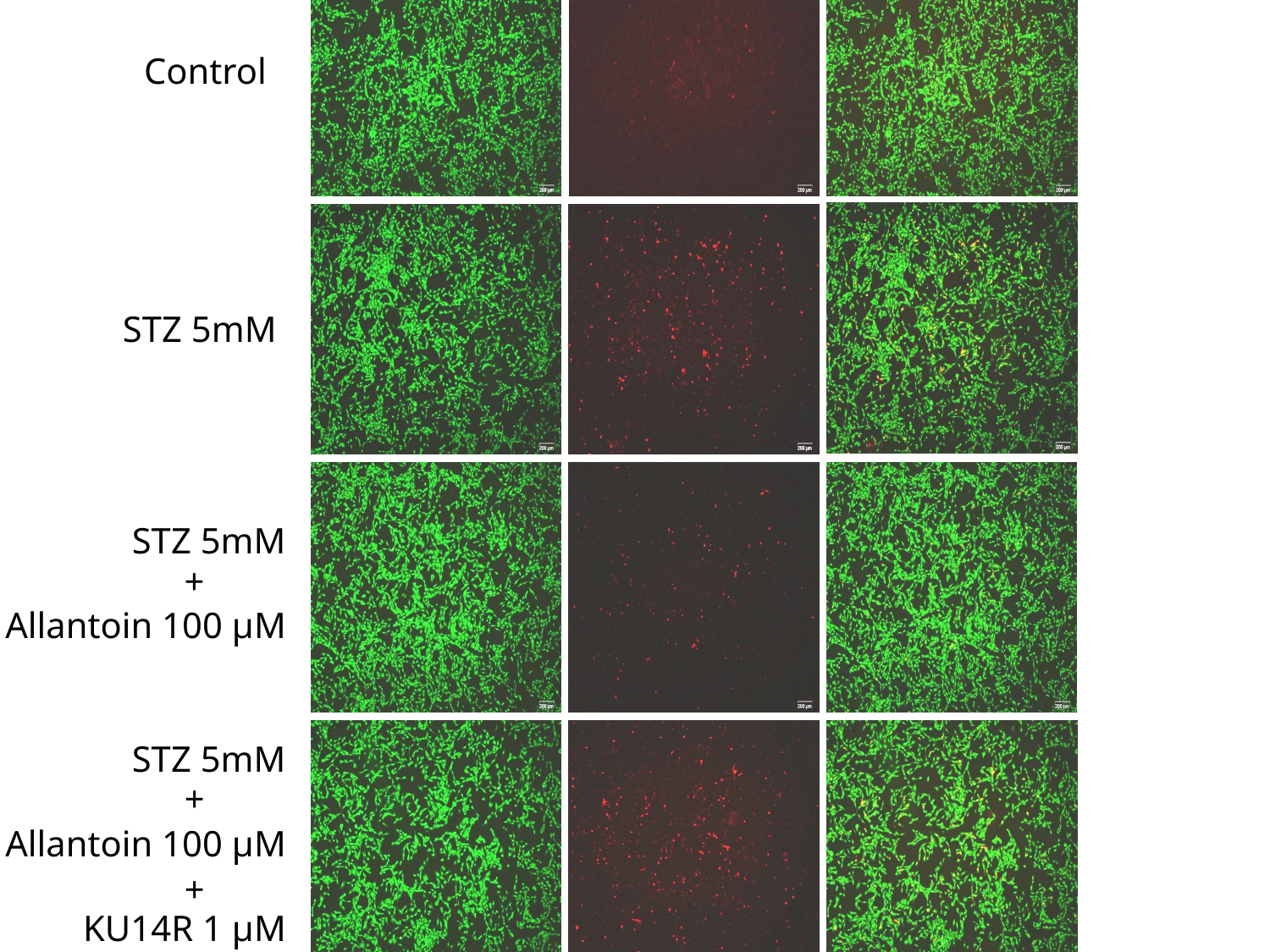

Live cells
Dead cells
Merge
Control
STZ 5mM
STZ 5mM
Allantoin 100 μM
+
STZ 5mM
Allantoin 100 μM
KU14R 1 μM
+
+

## Slide 6
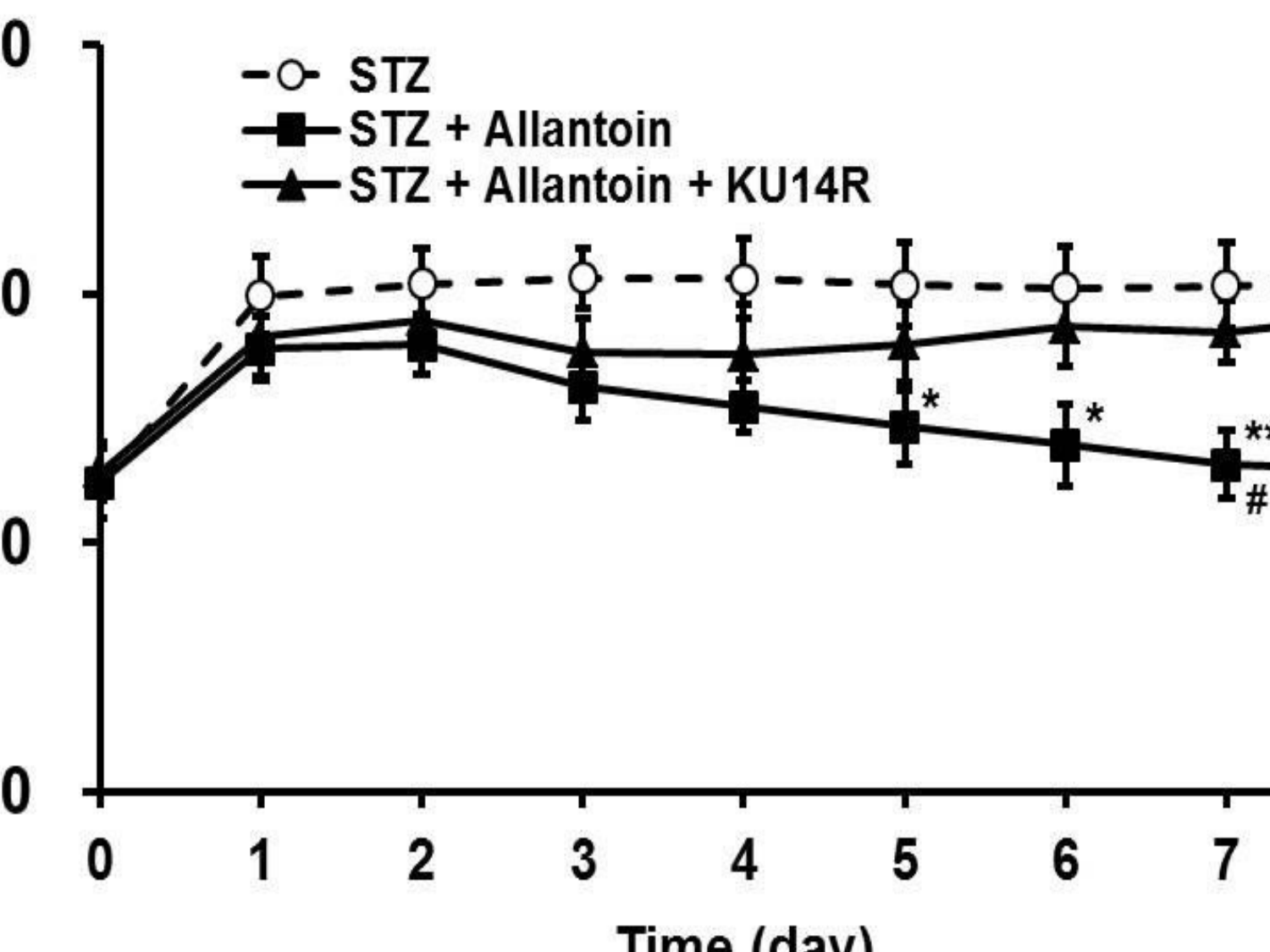

## Slide 7
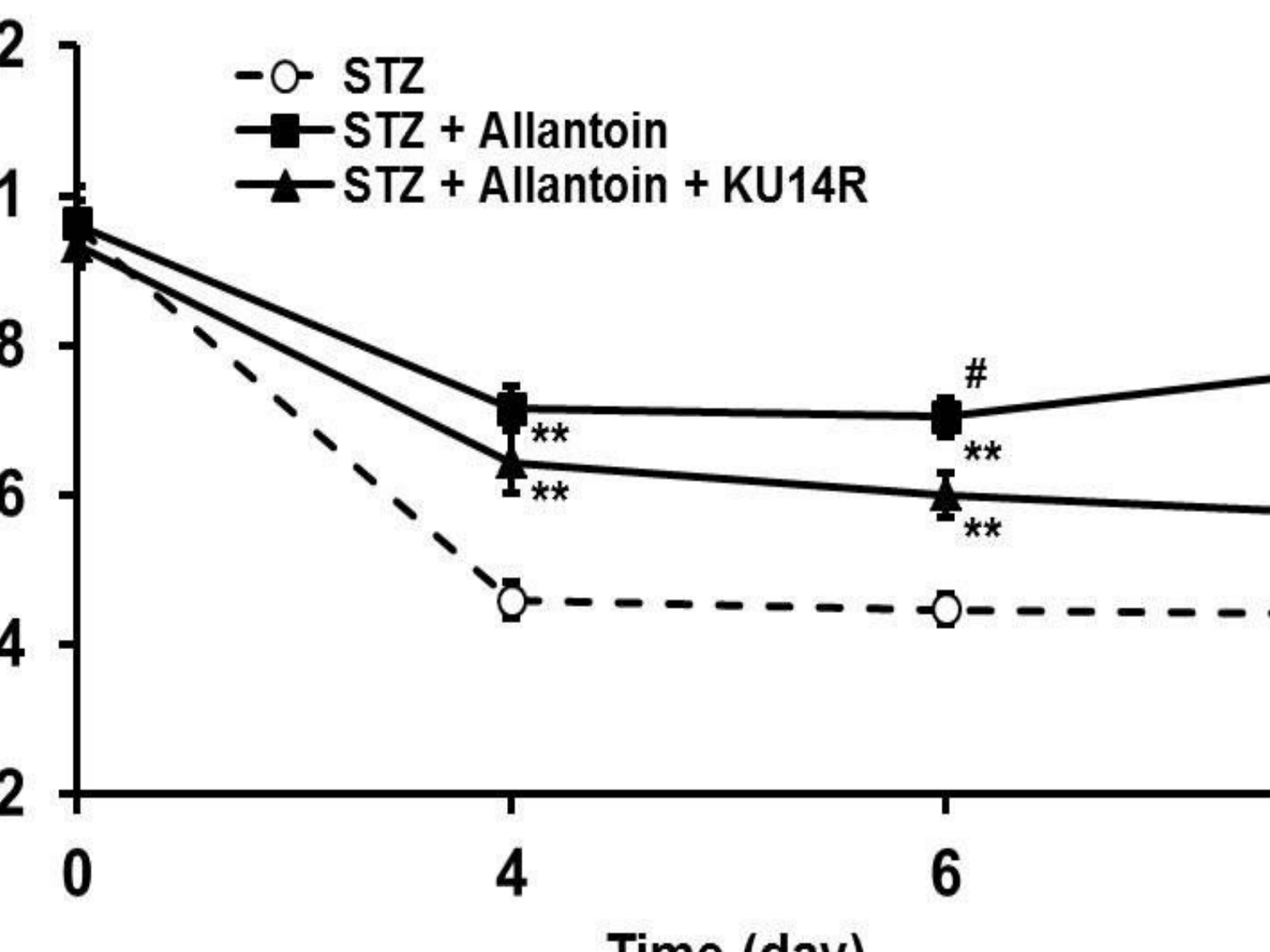

## Slide 8
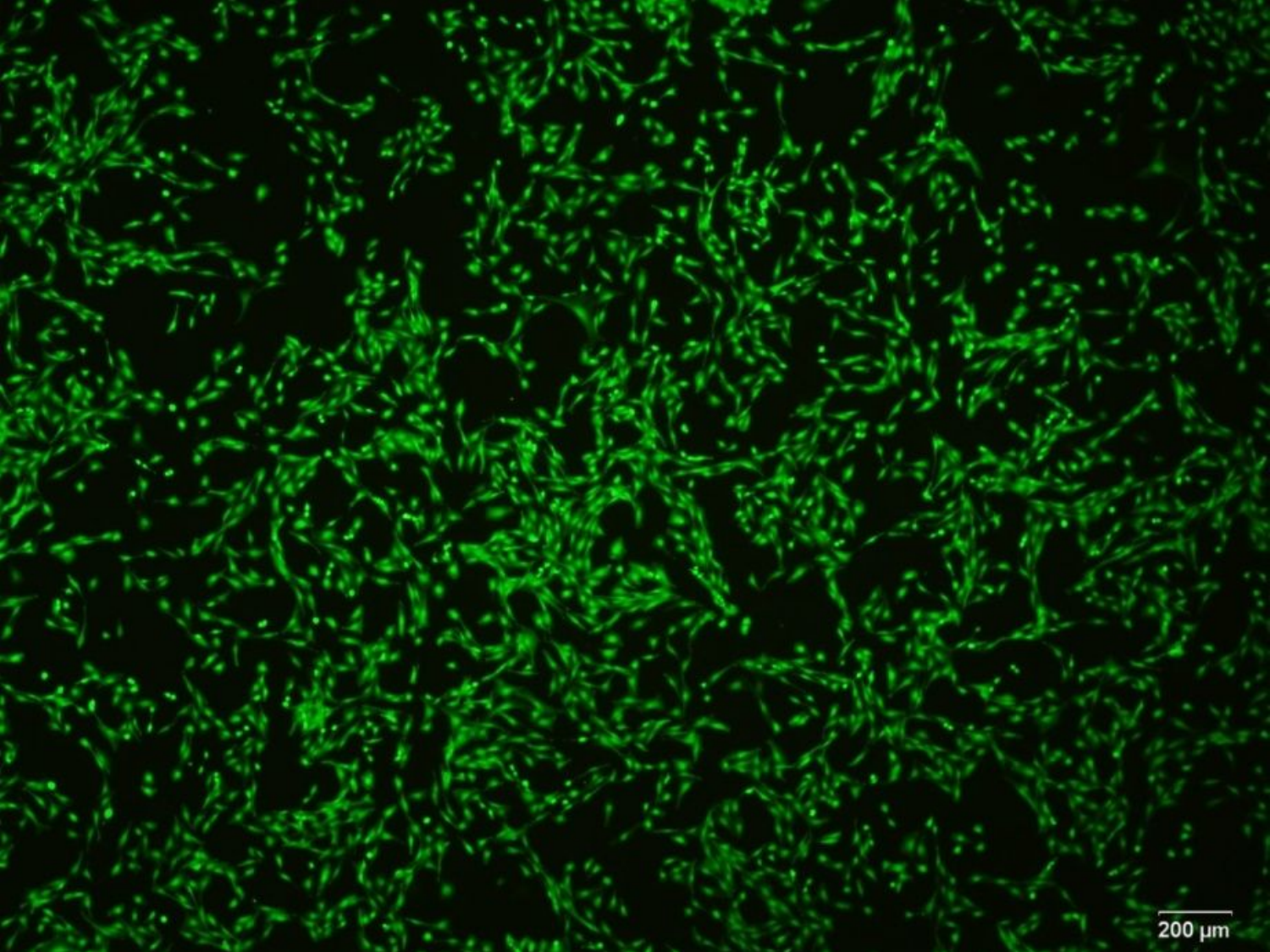

## Slide 9
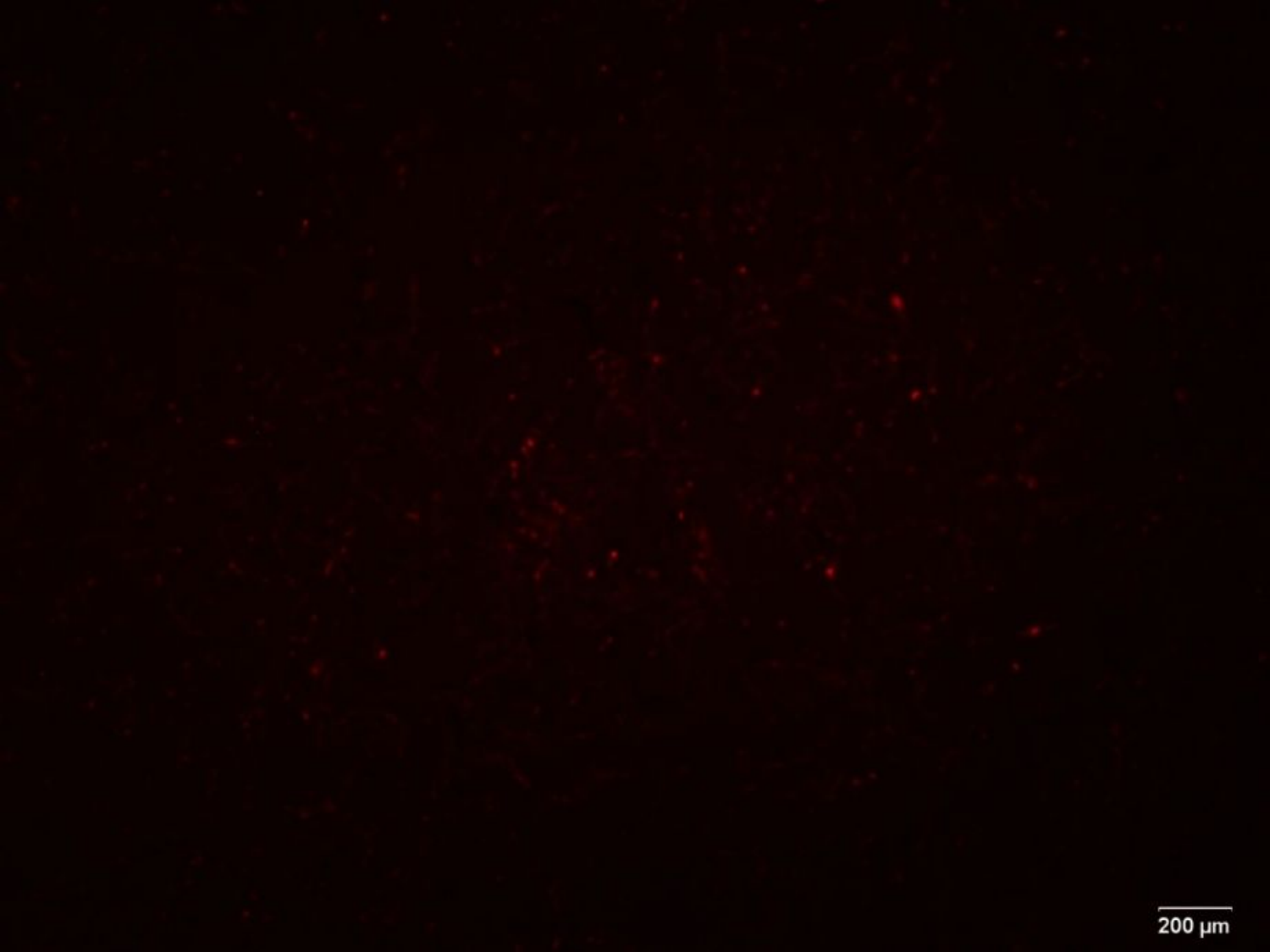

#

## Slide 10
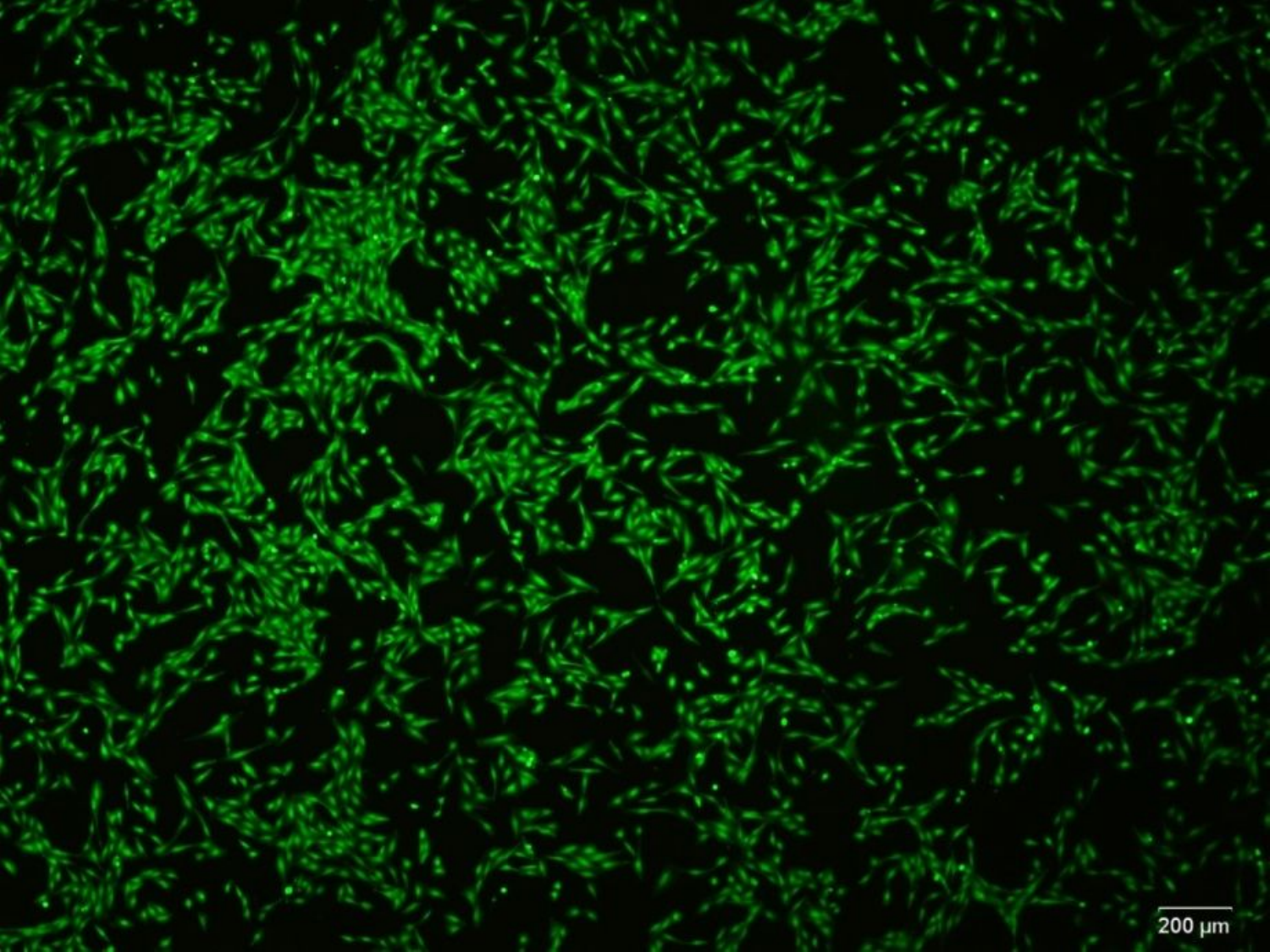

## Slide 11
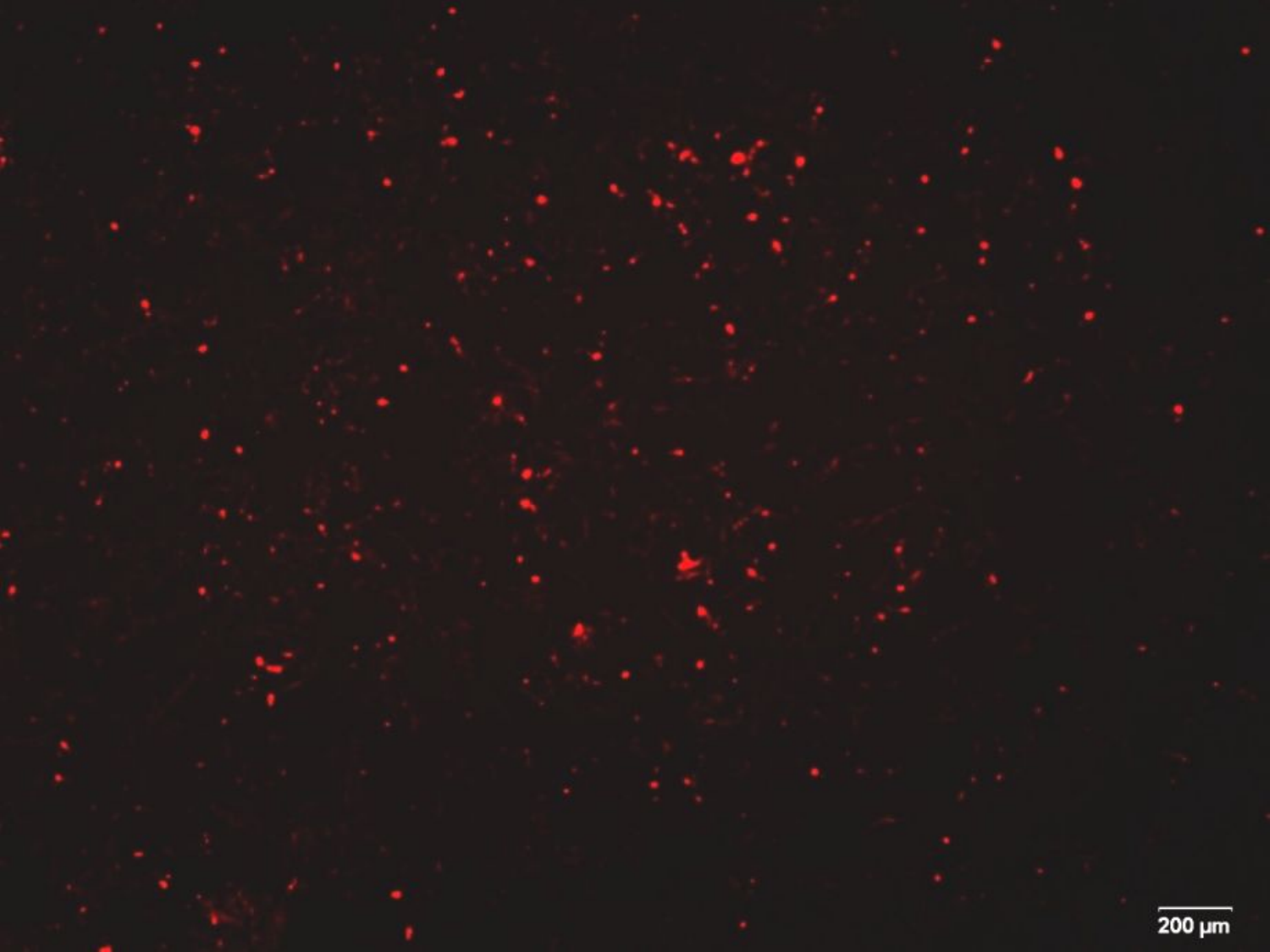

## Slide 12
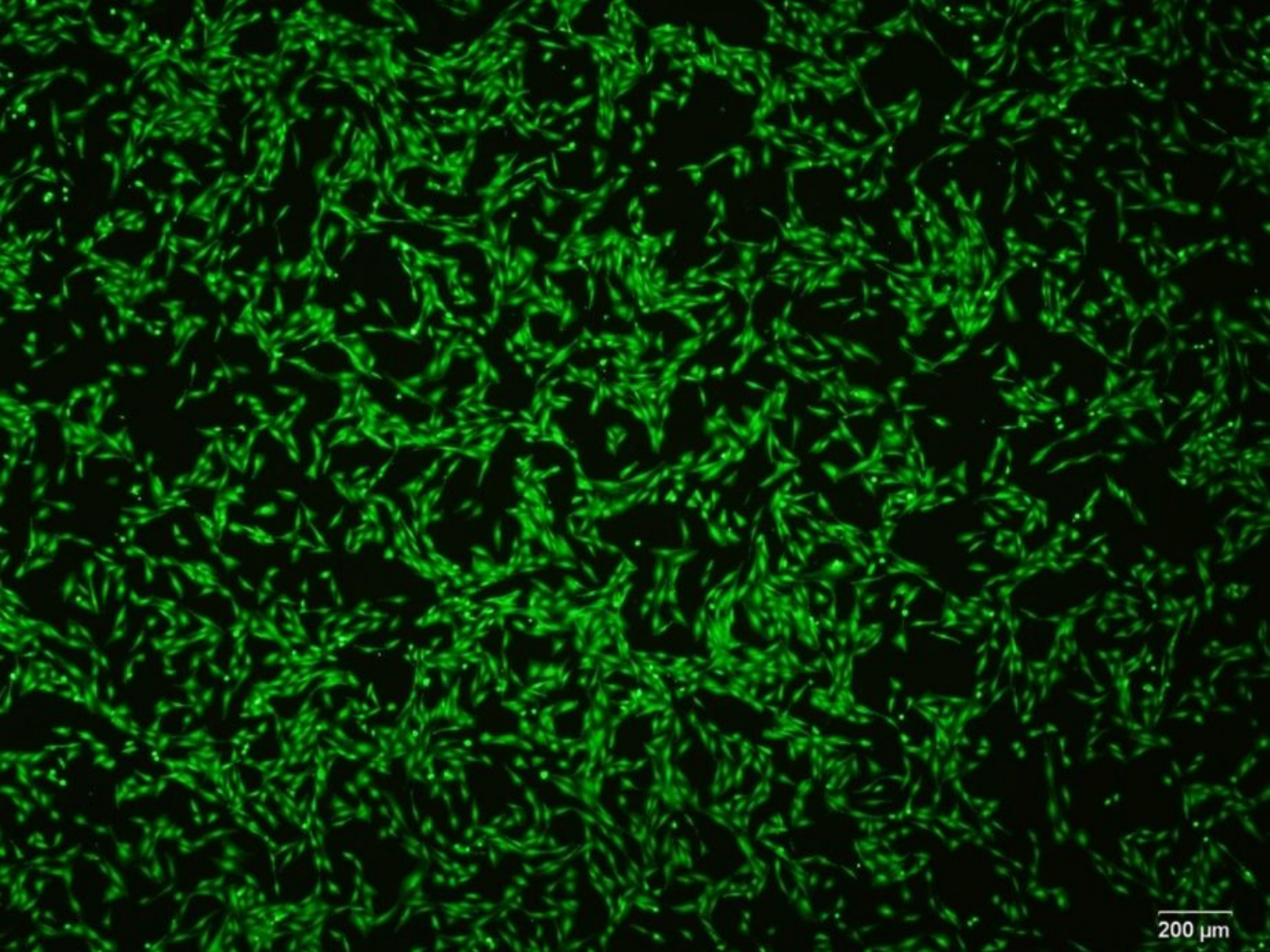

## Slide 13
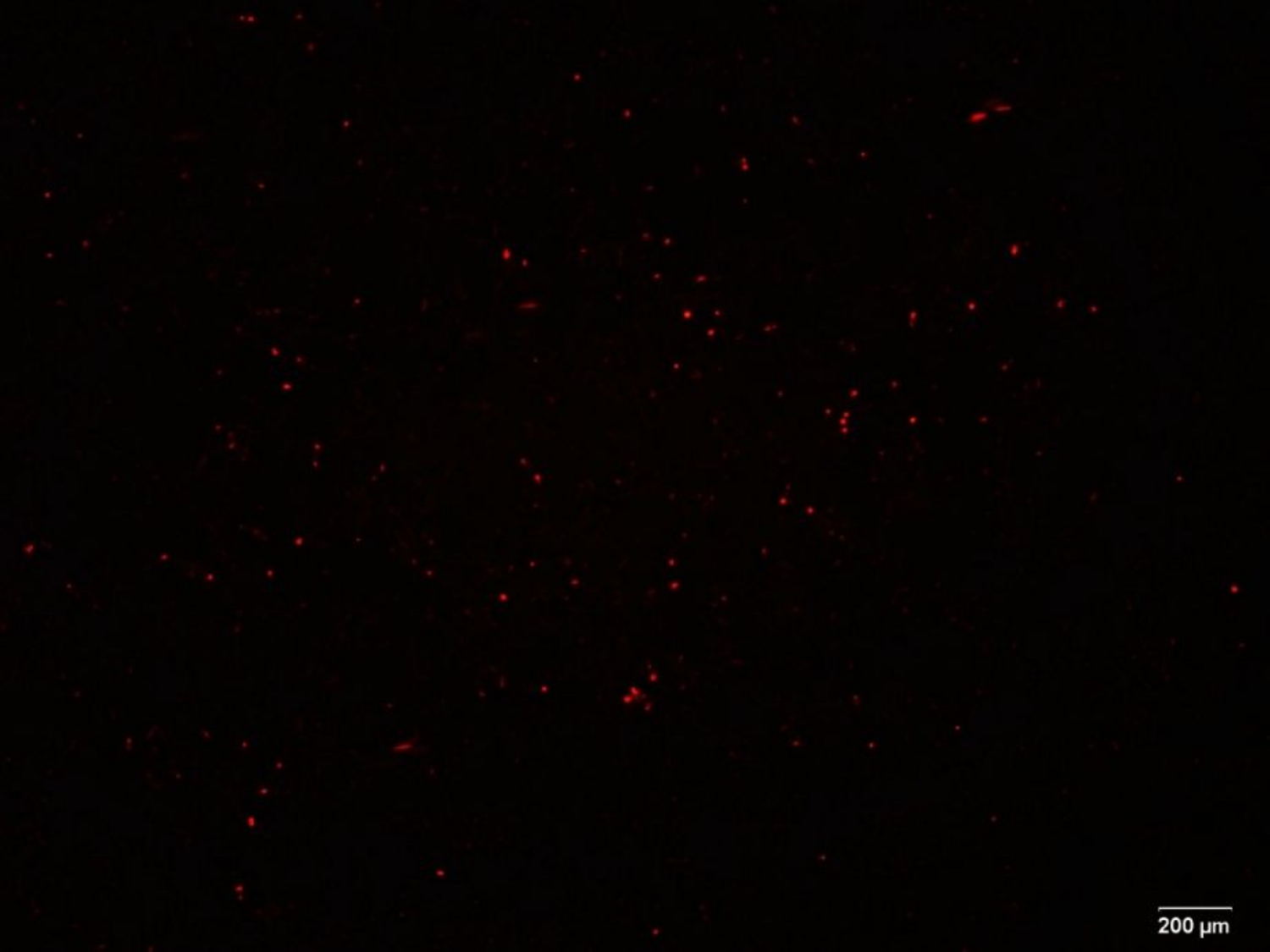

## Slide 14
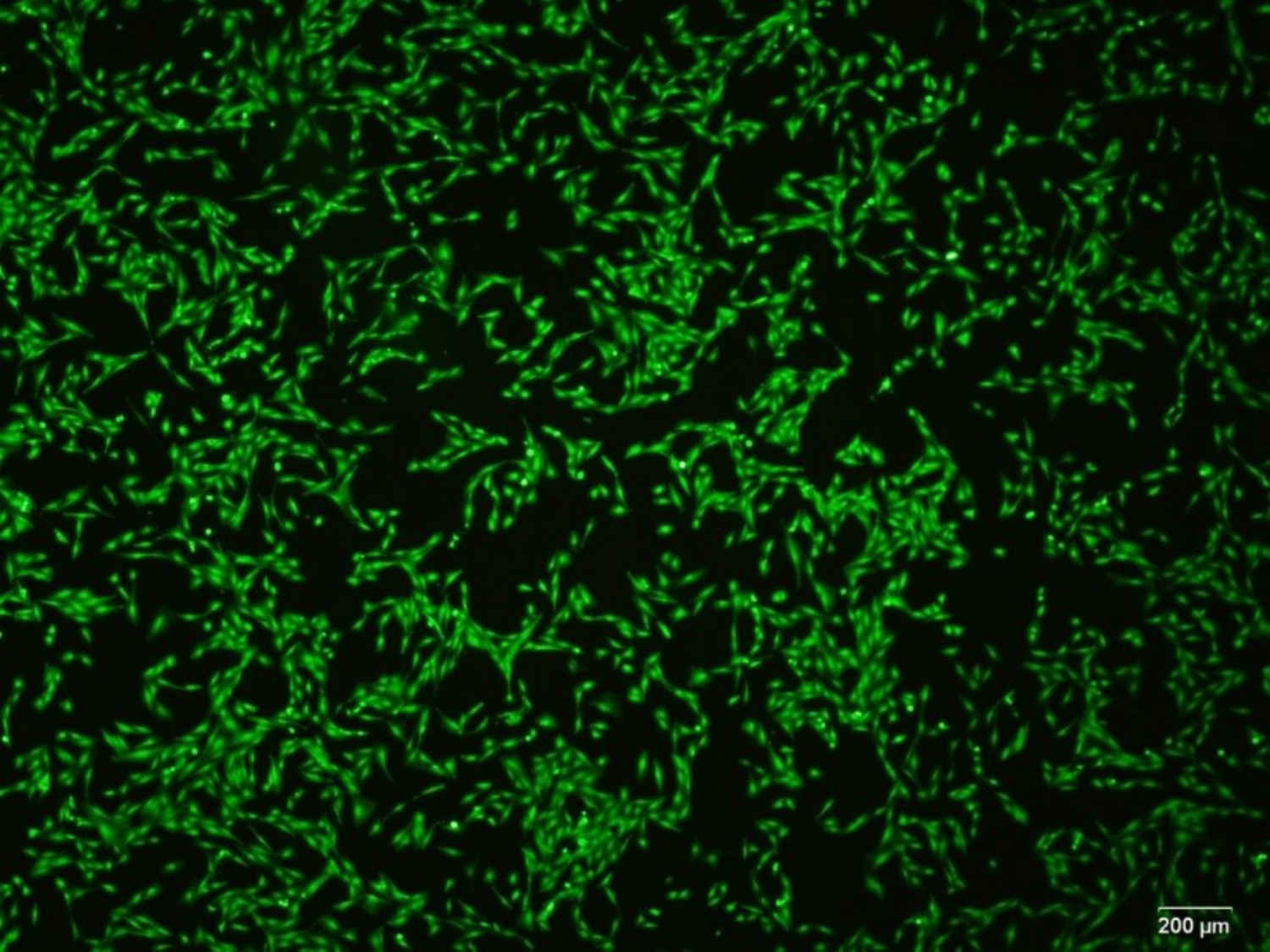

## Slide 15
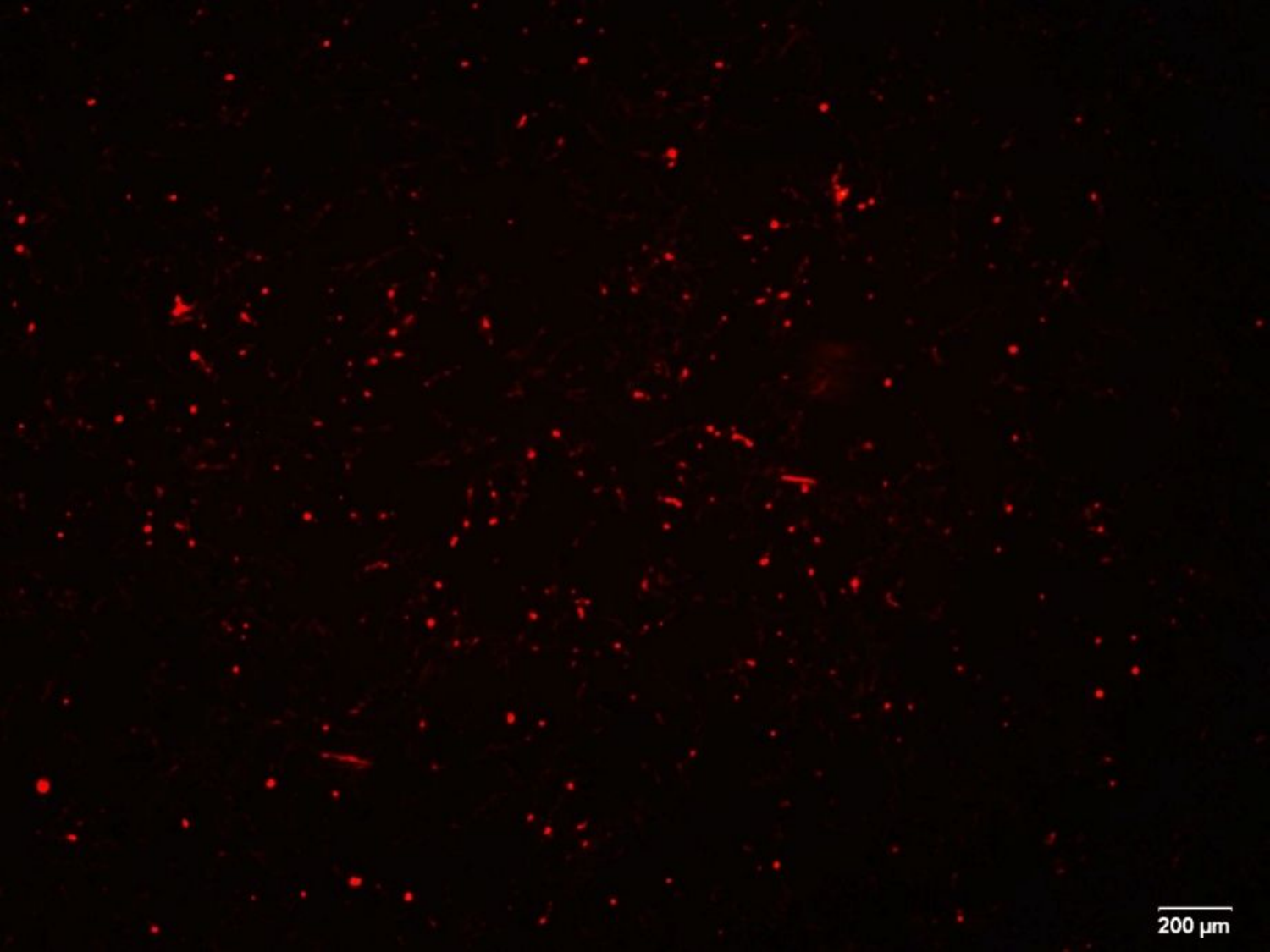

Supplement: Supplemental Information 1 [file peerj-03-1105-s001.zip › Allantoin raw data/Allantion live and dead.pptx]
