# Supplementary figures and images for: Allantoin ameliorates chemically-induced pancreatic β-cell damage through activation of the imidazoline I3 receptors
Source: PeerJ. 2015 Aug 6;3:e1105. doi: 10.7717/peerj.1105 (PMC4540048; doi:10.7717/peerj.1105)

## Slide 1
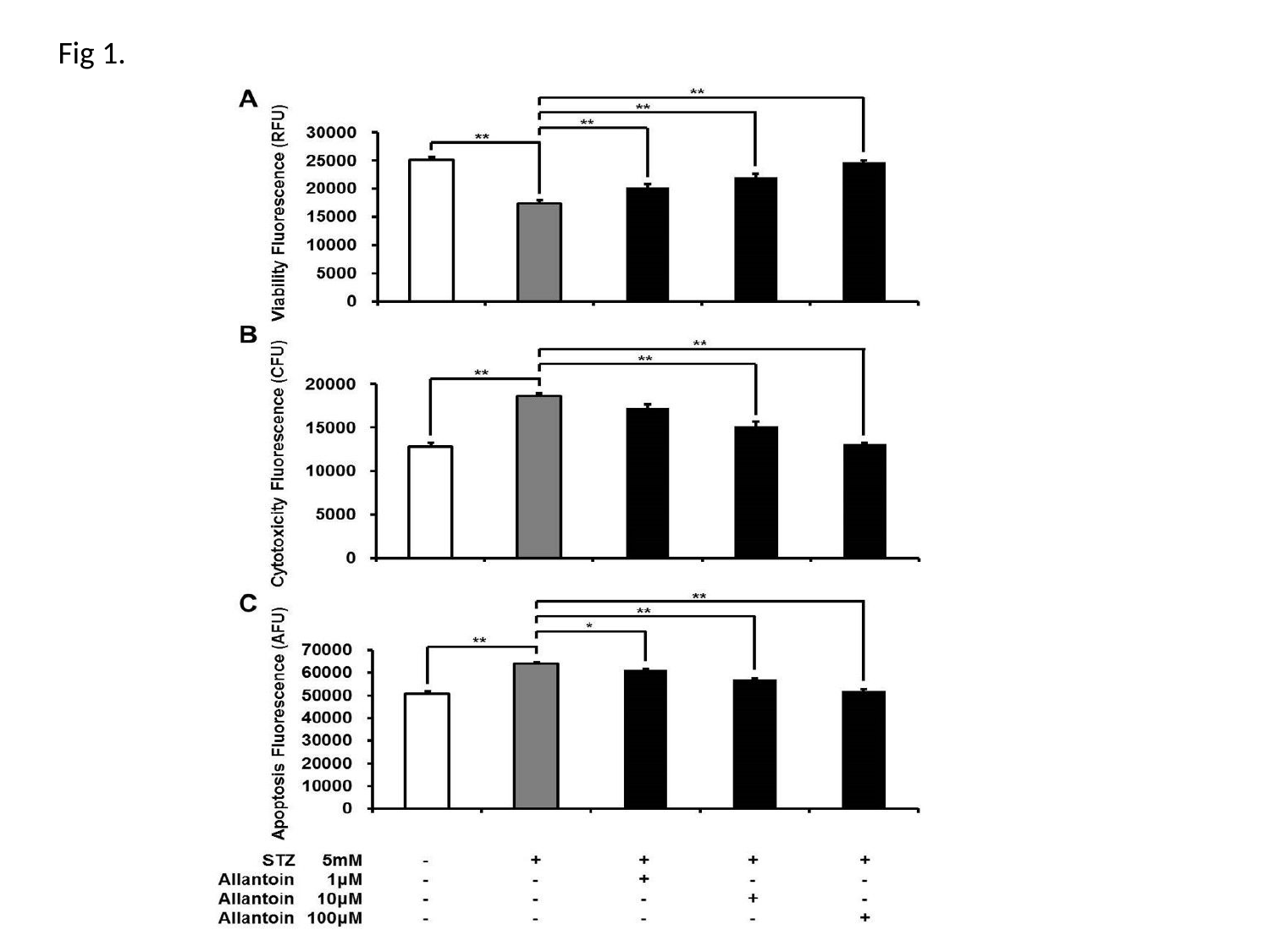

Fig 1.

## Slide 2
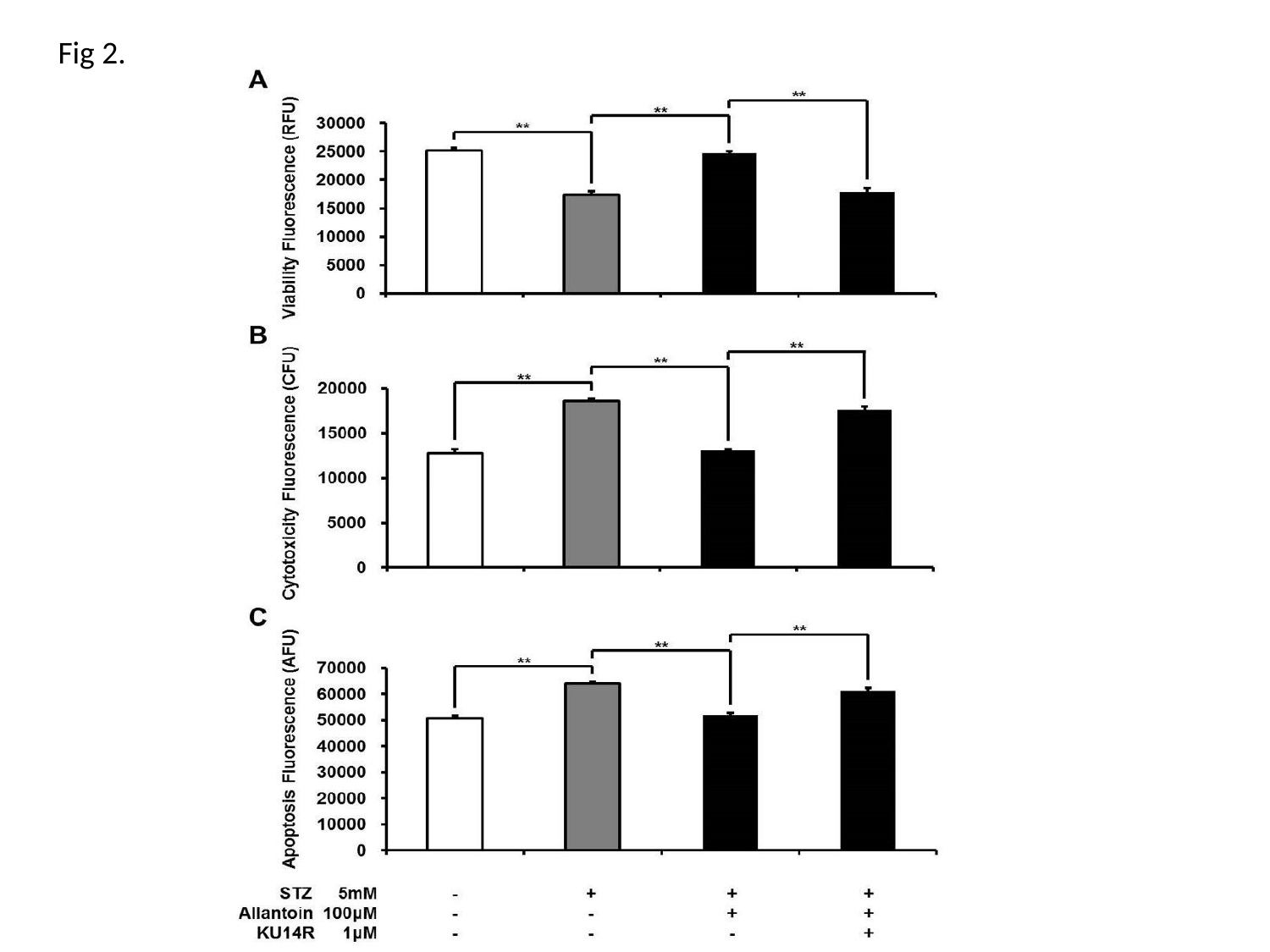

Fig 2.

## Slide 3
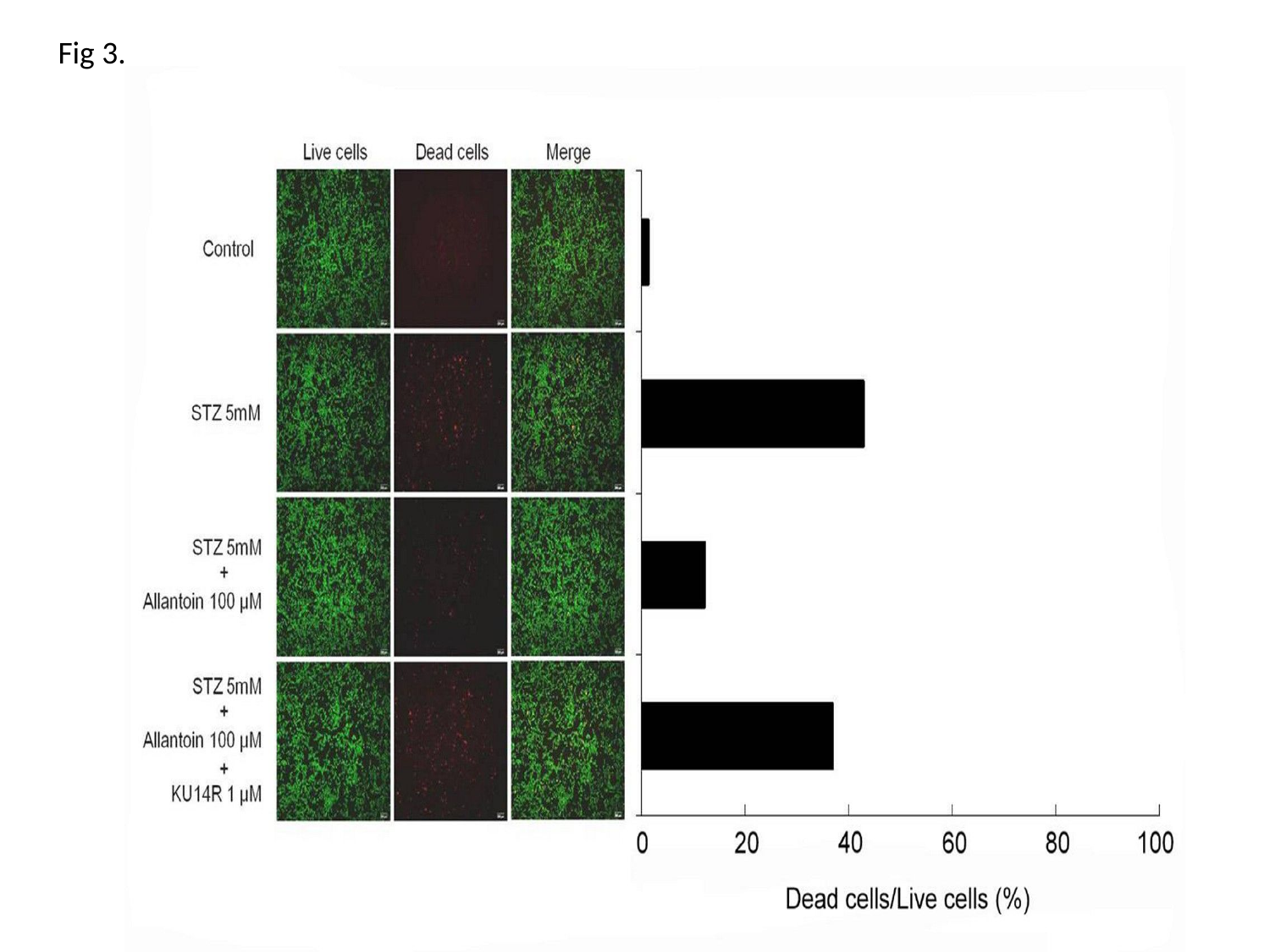

Fig 3.

## Slide 4
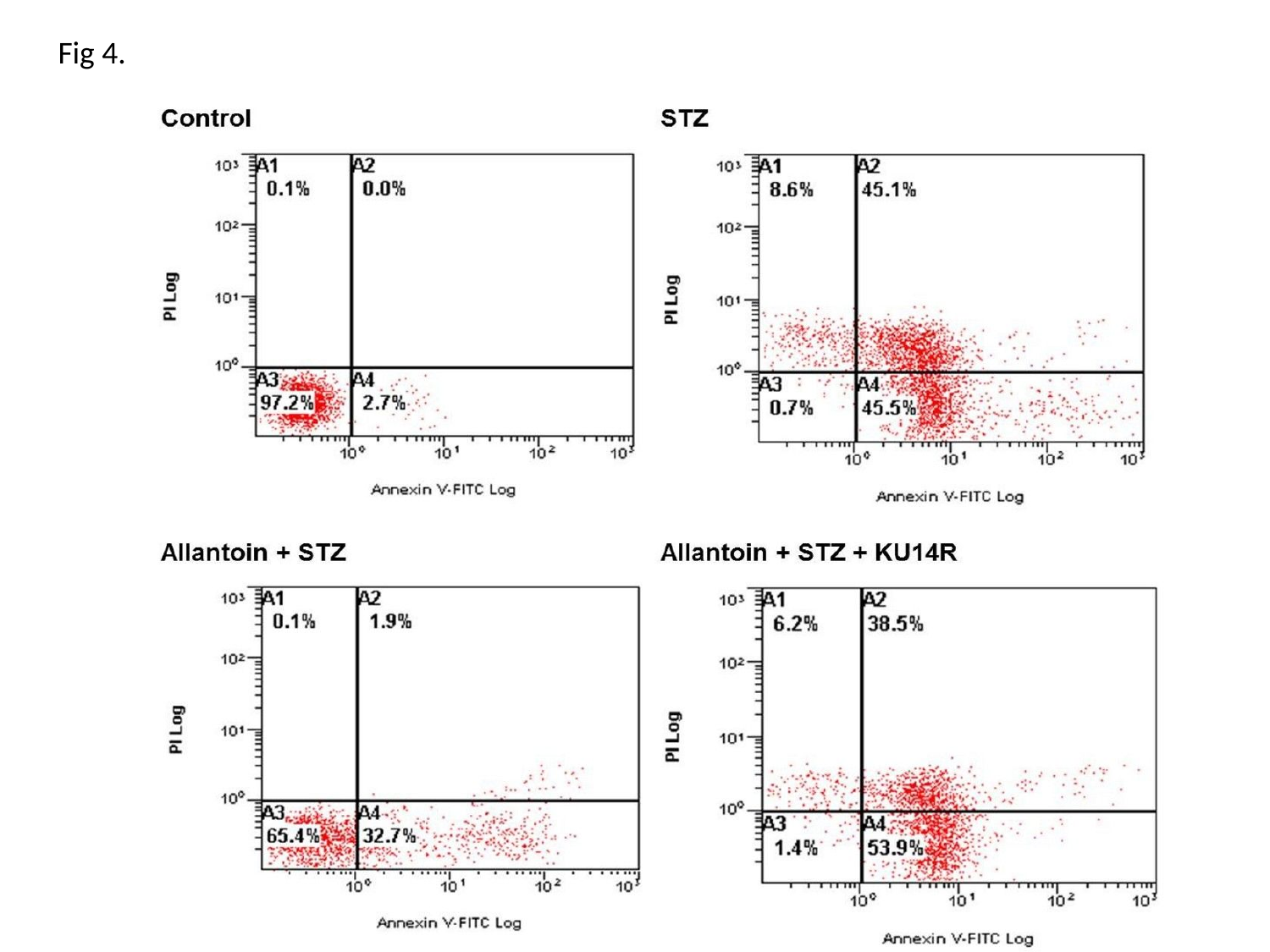

Fig 4.

## Slide 5
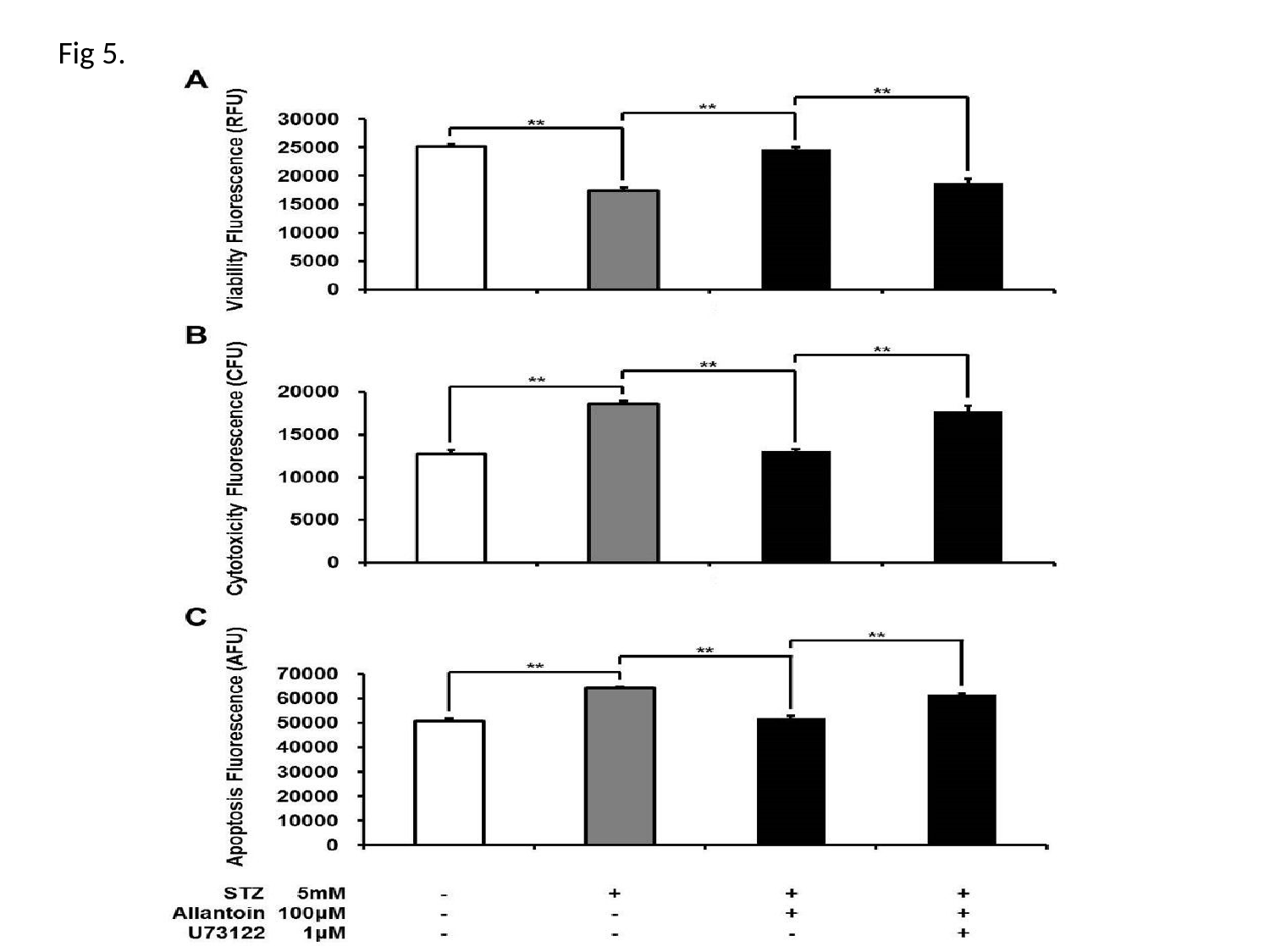

Fig 5.

## Slide 6
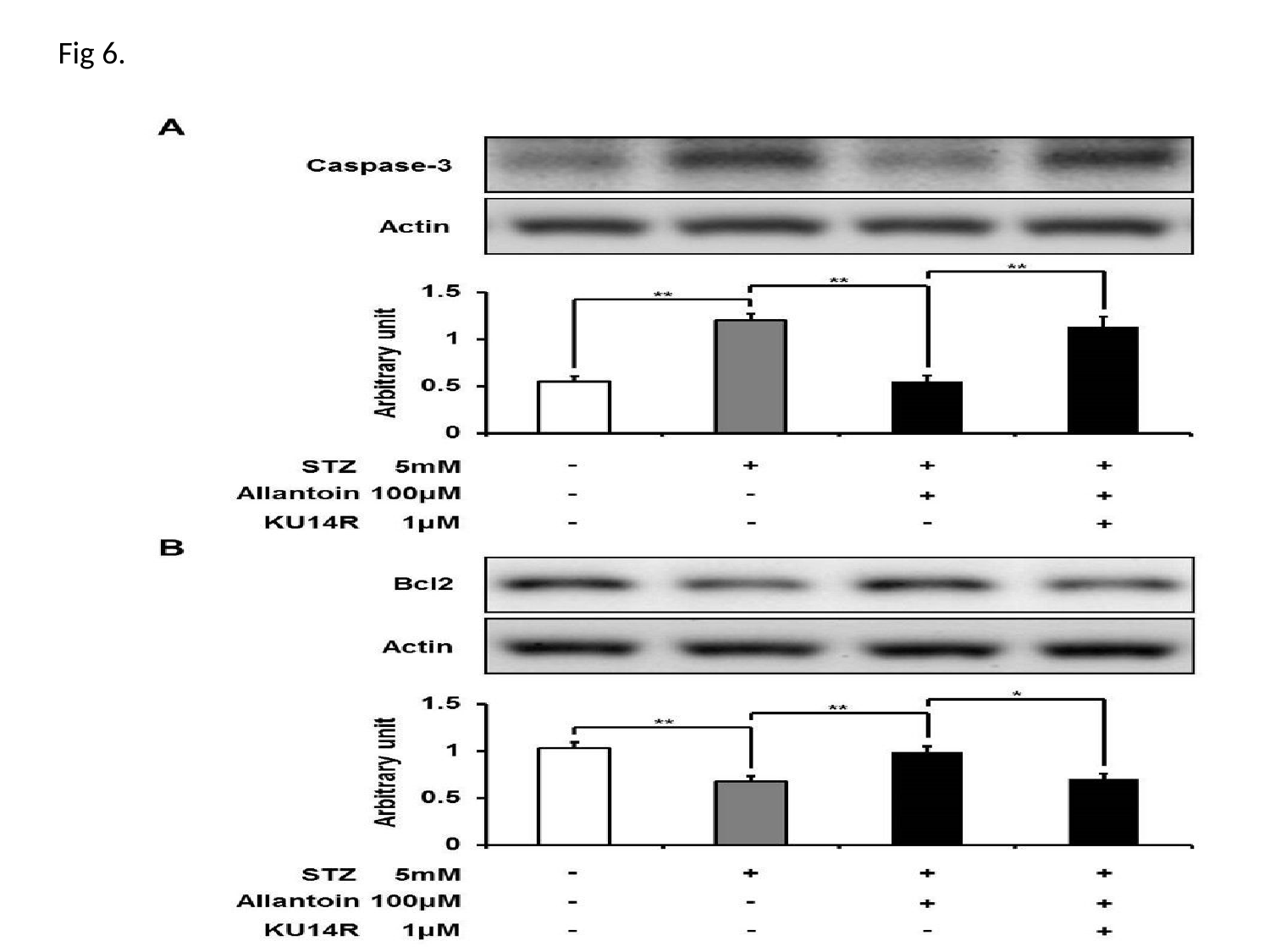

Fig 6.

## Slide 7
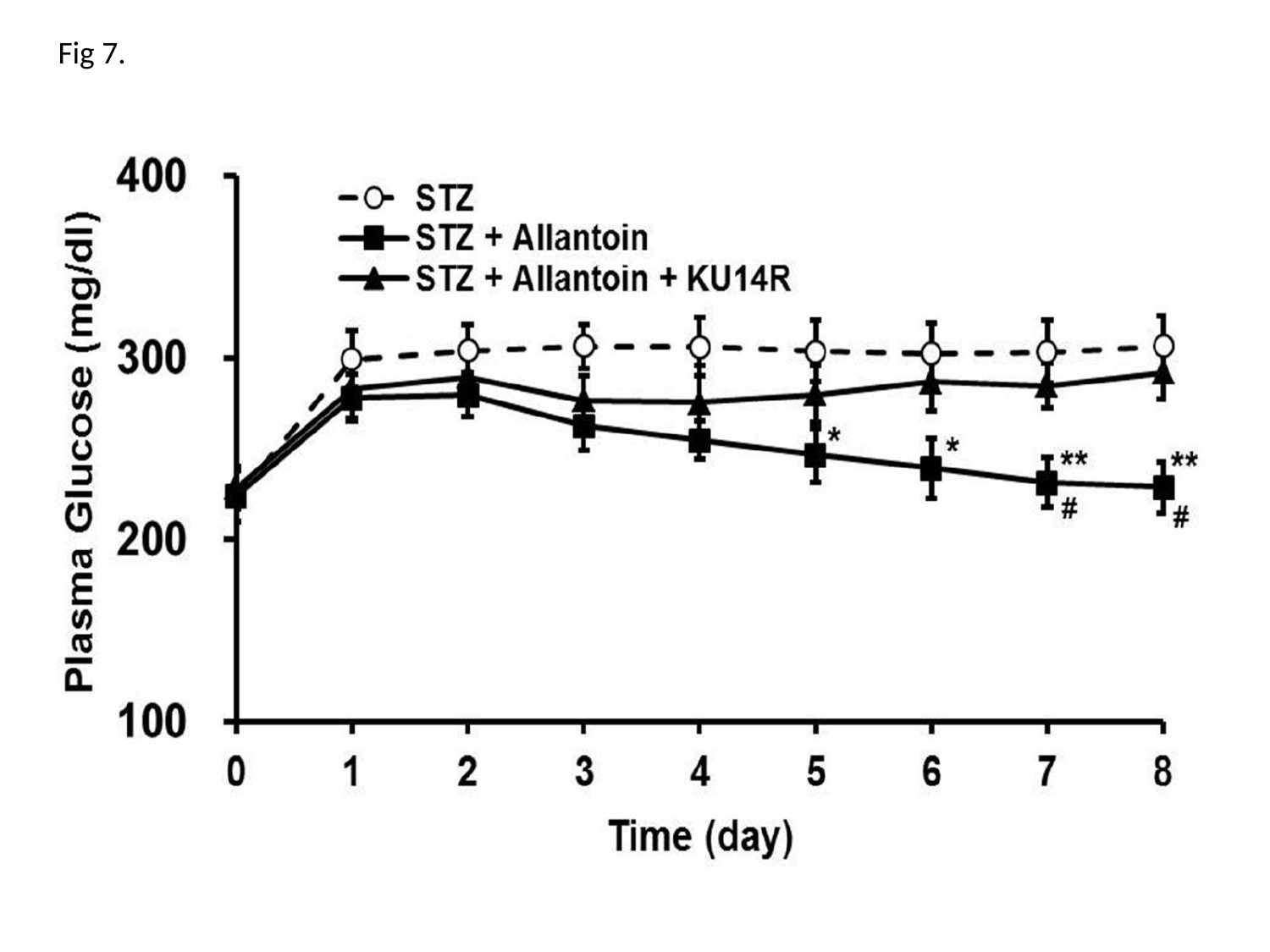

Fig 7.

## Slide 8
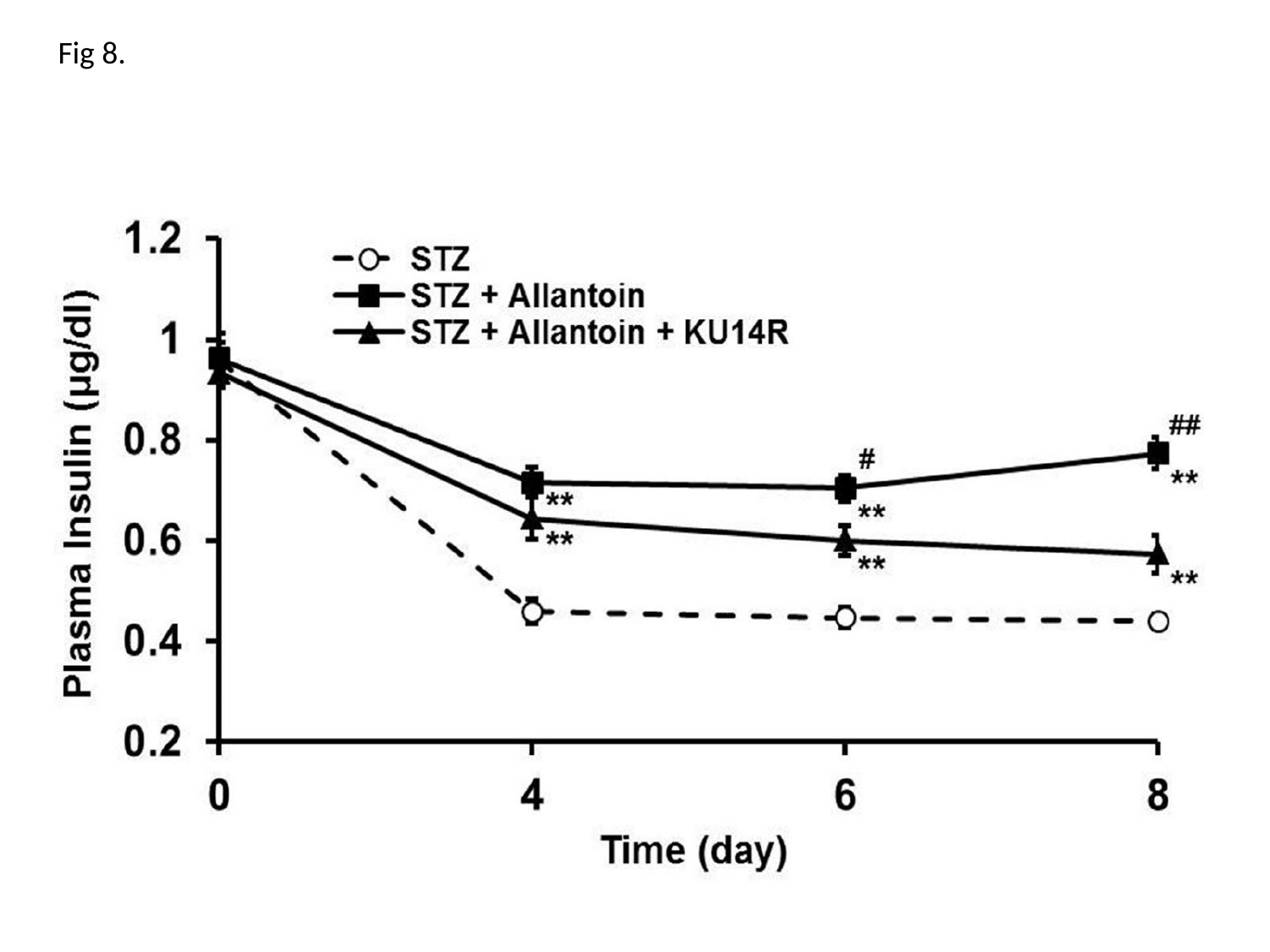

Fig 8.

Supplement: Supplemental Information 1 [file peerj-03-1105-s001.zip › Allantoin raw data/Final figure checked.pptx]
